# Impact of LASER activated irrigation on the retrievability of Guttaflow bioseal (in-vitro study)

**DOI:** 10.1038/s41405-024-00254-z

**Published:** 2024-09-11

**Authors:** Maram Obeid, Mohamed El Sayed Zaghloul, Tariq Yehia Abdelrahman

**Affiliations:** Endodontic Department, Faculty of Dentistry, Ain Shams University, Abassia, Cairo Egypt

**Keywords:** Root canal treatment, Dental lasers

## Abstract

**Background:**

This study aimed to evaluate the efficiency of diode LASER activated irrigation in the removal of GuttaFlow Bioseal root canal filling material during retreatment.

**Materials and subjects:**

Root canals of forty-five single-rooted human mandibular premolar teeth were prepared with ProTaper Universal rotary system and obturated with lateral condensation obturation technique using Gutta Percha and Roeko GuttaFlow Bioseal root canal sealer. All specimens were retreated with ProTaper Universal Retreatment System files then divided to three different groups according to the technique of activation of irrigation. Samples were sectioned, and the residual filling remnants were captured using digital camera attached to microscope. Data was collected by three different interpreters, to eliminate the subjectivity of the process, using the ImageJ Software. The mean value of the data was obtained and evaluated statistically. The significance level was set at *P* ≤ 0.05.

**Results:**

The remaining filling materials in the canals irrigated with ultrasonic activation (6.17 ± 1.42 at coronal level, 10.93 ± 1.91at middle level, and 14.58 ± 2.23 at apical level) were less than these irrigated with LASER activation (15.87 ± 3.78 at coronal level, 21.28 ± 4.44 at middle level, and 27.06 ± 2.68 at apical level). Maximum amount of remaining filling materials was present in the canals irrigated with passive side-vented syringe (23.07 ± 3.22 at coronal level, 38.09 ± 7.27 at middle level, and 34.24 ± 9.77 at apical level).

**Conclusion:**

The activation of irrigation techniques used were incapable of complete removal of filling material at root canal walls.

## Background

**M**anagement of root canal treatment failures is one of the most important scopes of Endodontology. The complete removal of gutta-percha and sealers is necessary to allow effective cleaning and refilling of the root canal system. However, complete removal is not always possible. Various irrigation techniques could be utilized in chemicomechanical removal of root canal sealer [1].

Bioceramic sealers (BCS) are biocompatible, bioinert, and dimensionally stable [2]. Upon setting, they produce hydroxyapatite which is osteoconductive and stimulates tissue regenerative responses [3]. On the other hand, bioceramic materials are known to be hard upon setting, the ability to retreat canals obturated with BCS is a current concern for practitioners [4].

Activated irrigation has been used in endodontic re-treatment to increase reduction of the volume of intracanal remnants through continuous movement of the irrigation solution. LASER-activated irrigation (LAI) has been introduced as a powerful method for root canal irrigation. The LASER radiation produces transient cavitation in the liquid through optical breakdown by strong absorption of the LASER energy. This allows more effective irrigation and further efficient cleaning [5].

No studies until now evaluated the efficacy of LASER activated irrigation in the removal of Guttaflow Bioseal root canal filling material during re-treatment. The null hypothesis stated that there is no difference between the LASER activated irrigation using diode LASER and the passive ultrasonic irrigation in removal of guttaflow bioseal root canal filling material during root canal retreatment.

## Materials and methods

### Selection and preparation of the specimens

1. Selection of teeth:
  Fourty-five freshly extracted mandibular premolars were used in the study. Sample size was calculated using power 80% and 5% significance level. Sample size calculation was achieved using G*Power software, Version 3.1.9.2. (Franz Faul, Kiel University, Germany).
  The selected teeth were radiographed in bucco-lingual and mesiodistal direction before preparation to observe pulp chamber and root canal system morphology. Teeth selected were all mature, single rooted mandibular premolars, with a single root canal and a length range between 18–22 mm and a mild angle of curvature (5–15 degrees). Teeth with immature roots or complex root canal anatomy, showing any detectable caries, cracks or resorption, were all excluded from the study.
2. Preparation of the teeth
  The selected teeth were thoroughly washed under running water to remove deposits on the root surface, access cavity was prepared in all samples under dental opertating microscope Seiler IQ (Seiler Instrument Inc., St. Louis, USA) at 7× magnification, using a high-speed diamond stone. The working length was then determined by introducing a size #10 k file (Mani Co., Kiyohara, Japan.) into the root canal until it became visible at the apical foramen confirming patency. The working length was established 1 mm short of that length. All teeth were prepared using Protaper Universal files (Dentsply Maillefer, Ballaigues, Switzerland) to file F5 with torque and speed settings adjusted according to the manufacturer’s instructions. The canals were irrigated with 2.6% NaOCl solution (Clorox Company, California, USA) throughout the preparation procedure after each file. When the instrumentation was completed, all canals were irrigated with 5 ml of 17% EDTA, followed by 5 ml of 2.6% NaOCl and 5 ml of sterile water. All irrigation process was done using irriflex (Produits Dentaires, St lmier, Switzerland) irrigation needles [4].
  Obturation was done using F5 gutta-percha (Dentsply Maillefer) with polydimethylsiloxane calcium silicate-containing sealer (Guttaflowbioseal, Coltene Whaledent, GmBH Co. KG, Langenau, Switzerland.) using cold lateral condensation technique [6]. All prepared specimens were stored at 37 °C in 100% humidity in an incubator for two weeks to allow complete setting of the sealer [1, 5].
3. Classification of specimens:
  The forty-five specimens were randomly divided into 3 experimental groups (each *n* = 15). Each group was retreated using Protaper Retreatment system (Dentsply Maillefer, Ballaigues, Switzerland) following manufacturer’s instructions [1]. The canal was then prepared using Protaper Universal F5 file at the full working length [1]. The canals were irrigated after using each file using different activation techniques according to assigned group.
  **Group I (G1)**: Irrigation was activated with Diode LASER (LAI):
    After each file, a total of 5 mL 2.6% NaOCl at room temperature was introduced into the canal and was activated using the 980 nm Diode LASER (Guilin Woodpecker Medical Instrument Co., Guangxi, China.) with the 200 μm fiber optic tip for a total of 20 s. For LAI, the LASER’s highest output was 5 watts at a 980 nm working wavelength. The LASER settings employed in this investigation were 1.2-watt power in pulsed mode. The tip was placed 1 mm short of the apex, activated, and then slowly pulled-out in a helicoidal movement at a speed of about 2 mm/sec touching the canal walls to promote even light diffusion inside the root canal lumen and to guarantee total irradiation of the canal’s wall from apical to coronal section [7]. The irradiation protocol was as follows: a lasing cycle consisted of a 5 s activation of irradiation, followed by a 20 s pause. For each tooth, the lasing cycle was applied four times, using 1.25 ml of 2.6% NaOCL each time. Radiation lasted for a total of 20 s [7]. After rinsing the canals with 2.5 ml distilled water, same protocol of irradiation was applied with a total of 5 ml of 17% EDTA. Thus, 1.25 mL of EDTA was used at each lasing cycle and the procedure was repeated four times. Consequently, the total radiation exposure for both irrigants was 40 s [7].
  **Group II (G2):** Irrigation was done using Conventional Passive side-vented Syringe Irrigation (CPI). A total of 5 ml of NaOCL and 5 ml of EDTA were used throughout the preparation. During each cycle the canals were irrigated after each file with 1.25 ml of 2.6% NaOCl at room temperature for 1 min followed by a 2.5 ml distilled water rinse, then a rinse with 1.25 ml of 17% EDTA solution for 1 min [7, 8].
  **Group III (G3):** Irrigation was activated using Passive Ultrasonic Activated Irrigation (PUI). The canals were irrigated after each file using 1.25 ml of 2.6% NaOCL at room temperature and activated with DBA S6 Ultrasonic Device (DBA, Guilin Woodpecker Medical Instrument CO., Guangxi, China) with Ultrasonic activation tip (ED60, Woodpecker DBA, China) being inserted 1 mm short of the WL into the canal for one minute. Thus, a total of 5 ml of 2.6% NaOCl was used during four cycles. After rinsing the canal with 2.5 mL of distilled water, the same PUI method was used to activate 5 ml of 17% EDTA at room temperature distributed on four cycles, each cycle 1.25 ml [7, 8].

Finally, the canals in all 3 groups were rinsed with 2.5 mL distilled water and dried with F5 paper points [5]. After that, samples were carefully sectioned vertically in buccolingual direction using a diamond disc until the shadow of the canal appeared through a thin layer of dentine then split longitudinally using a chisel into mesial and distal halves [1, 4]. The half of the roots that had the largest area of remaining obturation material were selected for scanning using digital microscope and [4] analyzed at coronal, middle and apical portions using a fixed magnification of 50X as follow: 0–3 mm from the apex for the apical portion, 3–6 mm from the apex for the middle portion, 6–9 mm from the apex for the coronal portion [4].

**Fig. 1:**
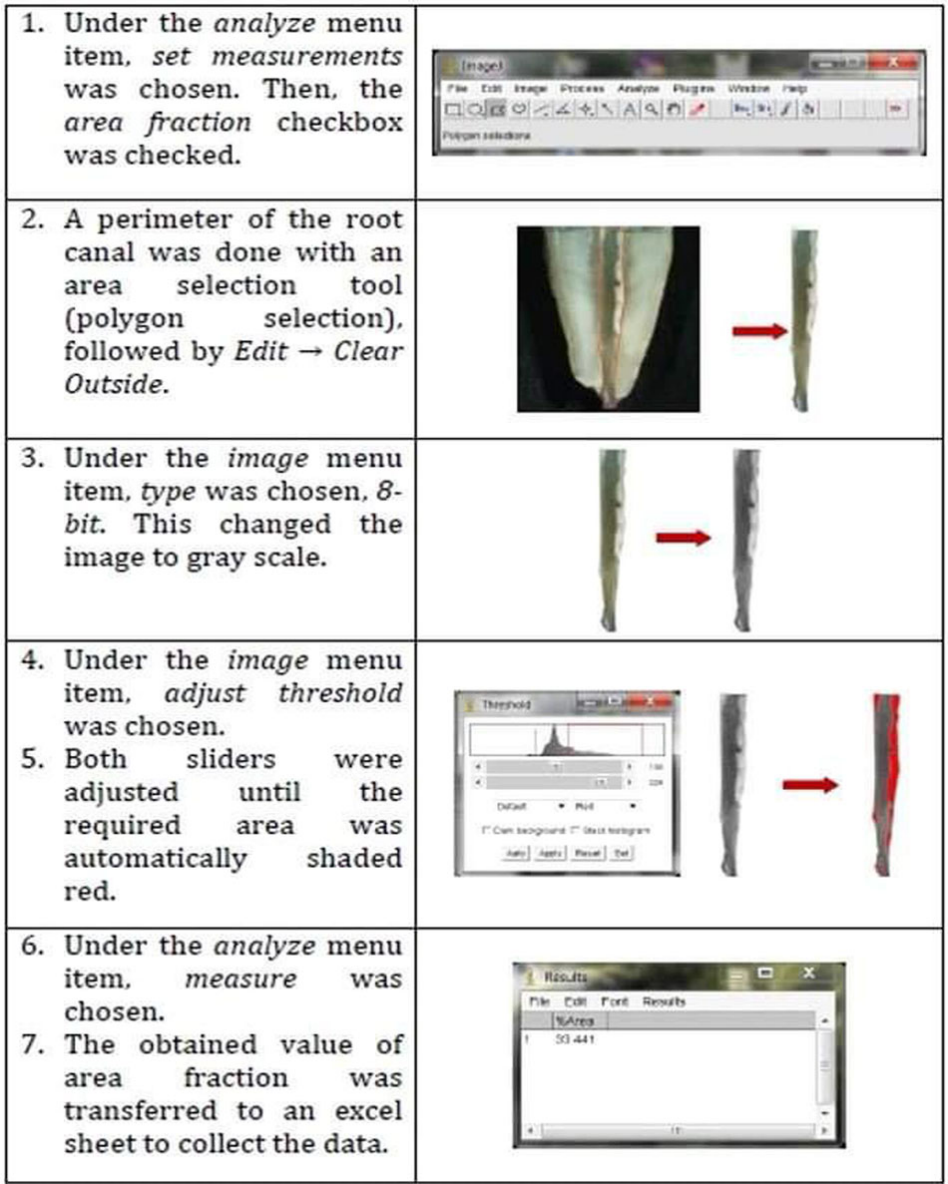
Photograph showing how ImageJ software was used to analyze and measure the percentage of area covered by remaining obturation material.

Images were captured using Dino-Lite digital camera attached to microscope, and then transferred to desktop and saved as JPEG format [1, 4, 7]. ImageJ software was used to analyze the obtained images by measuring the percentage of area covered by remaining obturation material [7] (Fig. 1).

Data was collected by three different interpreters using the ImageJ Software, to eliminate the subjectivity of the process [1]. The mean value of the data was obtained and was evaluated statistically. The mean and standard deviation values were calculated for each group in each test. Data were explored for normality using Kolmogorov–Smirnov and Shapiro–Wilk tests, data showed parametric normal distribution. The significance level was set at *P* ≤ 0.05.

## Results

**Fig. 2:**
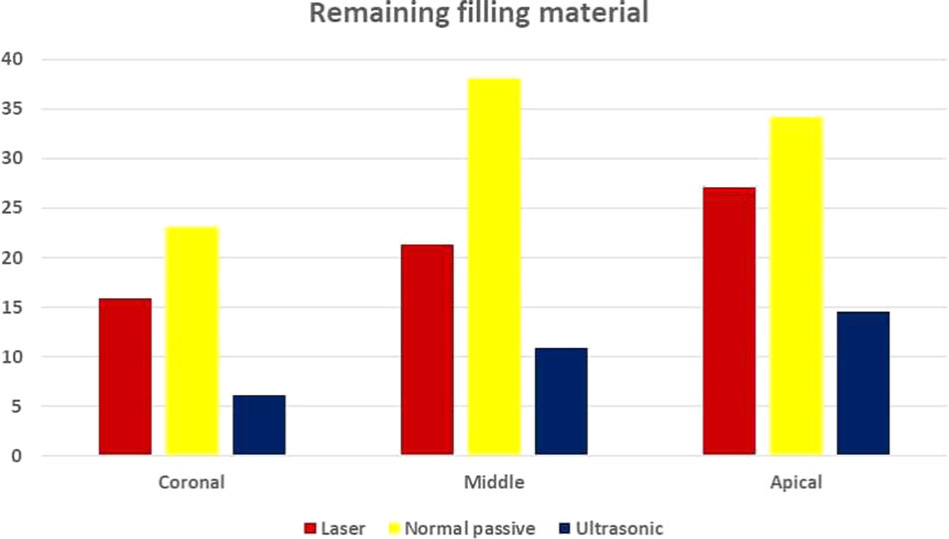
Bar chart showing the mean values of remaining filling material of different groups.

The results showed that the methods of irrigation activation had a statistically significant effect on the remaining material mean value (Table 1) (Fig. 2). Moreover, root canal thirds had a statistically significant effect. The interaction between the two variables also had a statistically significant effect.

**Fig. 3:**
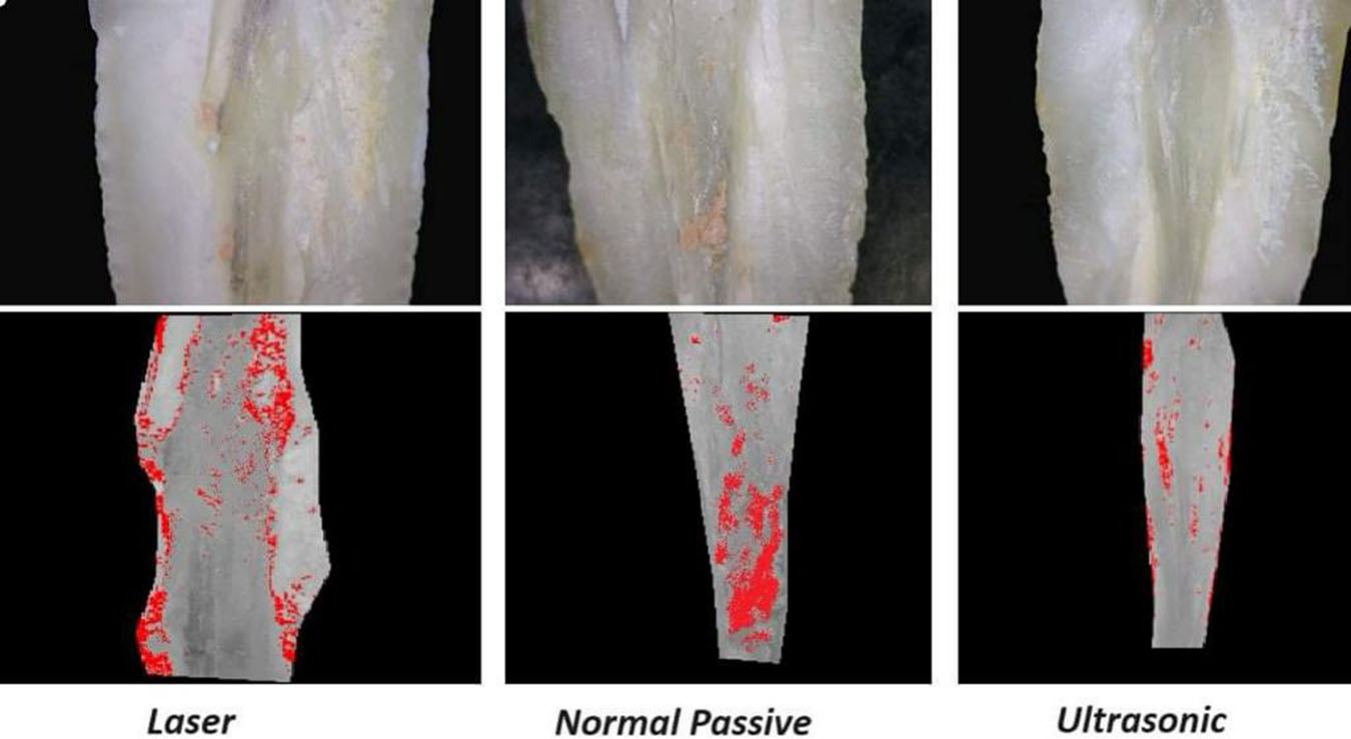
Digital micrograph showing remaining filling material of different groups at coronal third of root canals walls.

**Fig. 4:**
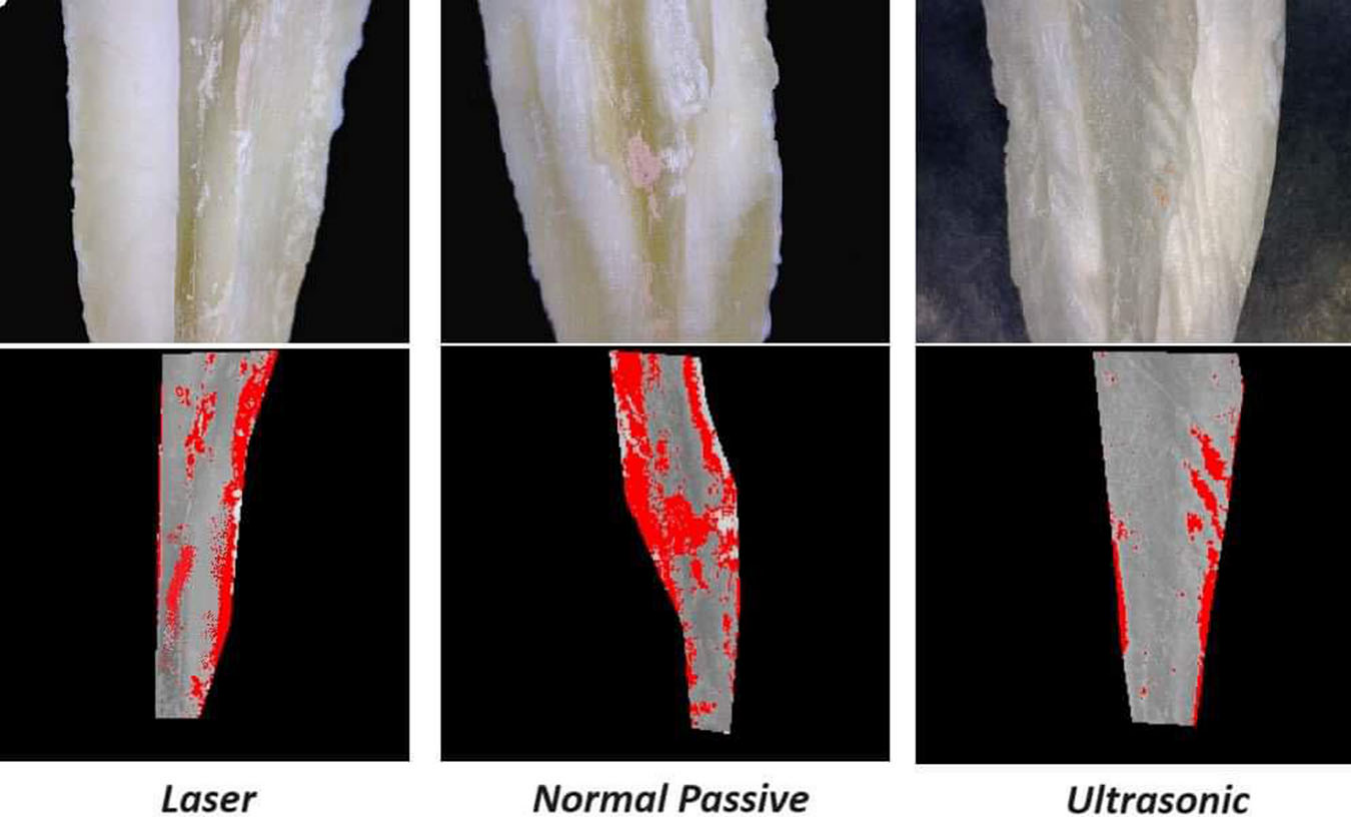
Digital micrograph showing remaining filling material of different groups at middle third of root canals walls.

A. **At coronal third of root canals wall:**
  The highest mean value was found in Conventional Passive group (23.07 ± 3.22) followed by the LASER group (15.87 ± 3.78) while the least mean value was found in the Ultrasonic group (6.17 ± 1.42). There was a statistically significant difference between LASER, Conventional Passive and Ultrasonic groups where (*P* < 0.001). A digital micrograph representing these data in Fig. 3.
B. **At middle third of root canals wall**:
  The highest mean value was found in Conventional Passive (38.09 ± 7.27) followed by LASER (21.28 ± 4.44), while the least mean value was found in Ultrasonic group (10.93 ± 1.91). There was a statistically significant difference between LASER, Conventional Passive and Ultrasonic groups where (*P* < 0.001). A digital micrograph representing these data in Fig. 4.
C. **At apical third of root canals wall:**

**Table 1:** The mean, standard deviation (SD) values of remaining filling material of different groups.

Variables	Remaining filling material						
	LASER		Conventional Passive		Ultrasonic		*p*-value
	Mean	SD	Mean	SD	Mean	SD	
Coronal	15.87^cB^	3.78	23.07^cA^	3.22	6.17^cC^	1.42	<0.001*
Middle	21.28^bB^	4.44	38.09^bA^	7.27	10.93^bC^	1.91	<0.001*
Apical	27.06^aB^	2.68	34.24^aA^	9.77	14.58^aC^	2.23	<0.001*
*P-value*	<0.001*		0.001*		<0.001*		

Values with different superscript letters are significantly different. *Significant (*P* < 0.05) *ns* non-significant (*P* > 0.05).

**Fig. 5:**
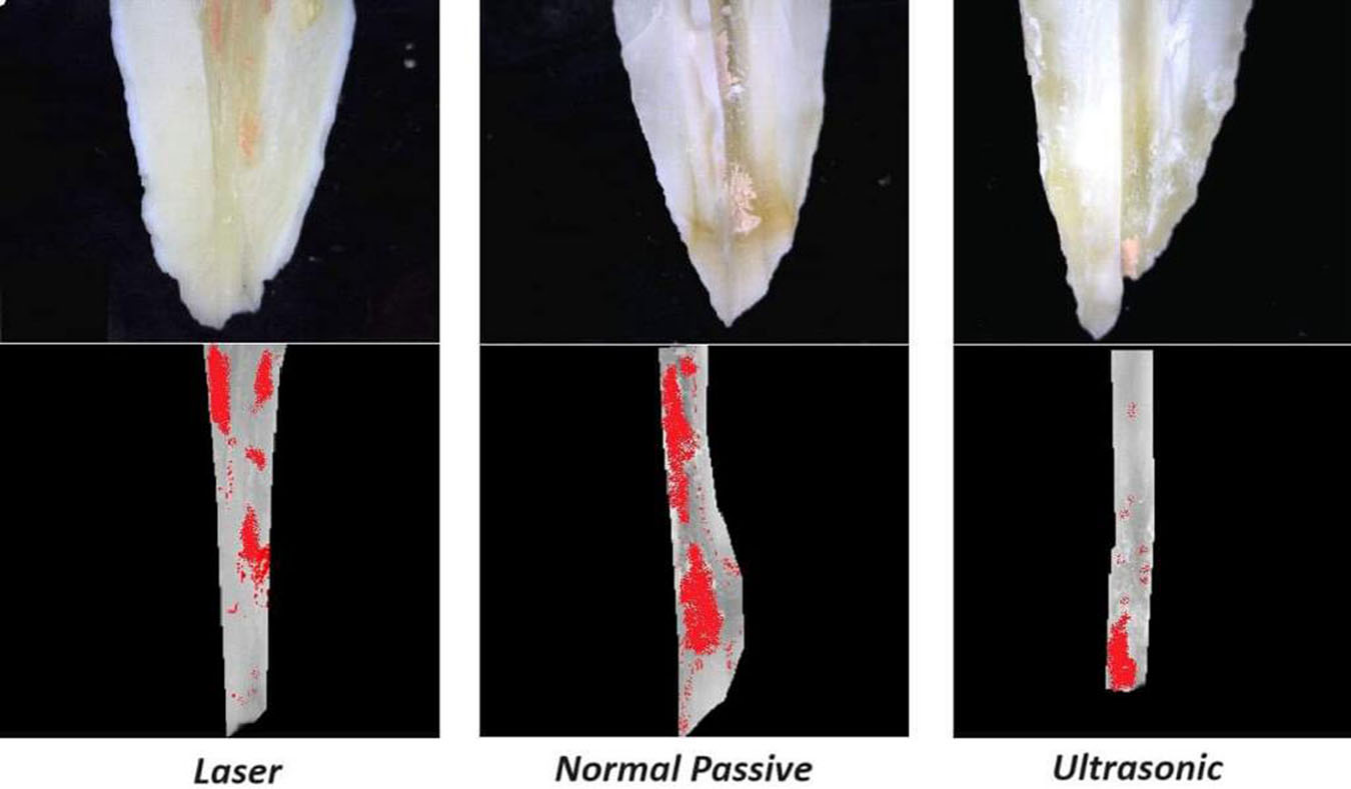
Digital micrograph showing remaining filling material of different groups at apical third of root canals walls.

The highest mean value was found in Conventional Passive group (34.24 ± 9.77) followed by LASER (27.06 ± 2.68), while the least mean value was found in Ultrasonic group (14.58 ± 2.23). There was a statistically significant difference between LASER, Conventional Passive and Ultrasonic groups where (*P* < 0.001). A digital micrograph representing these data in (Fig. 5).

## Discussion

**N**on-surgical endodontic retreatment has been the chosen treatment for endodontic treatment failure [9, 10]. Removal of old obturation material is essential in endodontic re-treatment. Infected debri with remaining filling material, can be the main cause for persistent periapical pathosis. Therefore, the maximum quantity of old obturation material should be removed to allow proper disinfection of the root canal system [9, 10].

The most commonly used core material for root canal filling is gutta-percha. Unfortunately, owing to its poor sealing properties a sealer should be used in combination with Gutta-percha [11]. The sealer has a critical role in sealing the canal space by filling-up the irregularities between the canal wall and the core filling material [12]. Recently, new polydimethylsiloxane (silicon)-based sealers are considered as viable alternative to calcium silicate-based sealer. They have clinical advantages such as homogeneity, viscosity and elasticity which allow them to adapt to stresses generated by mastication during root flexure [13]. These materials are marketed by Coltene Whaledent, such as: RoekoSeal Automix, GuttaFlow 2, and GuttaFlow bioseal [13].

GuttaFlow bioseal is a hydrophilic sealer with gutta-percha powder, polydimethylsiloxane, and bioactive glass ceramic. It has alkalinizing activity with calcium ions release and minimum solubility when compared to GuttaFlow 2 and RoekoSeal Automix [13]. According to Gandolfi et al. [14], the incorporation of a calcium silicate can be beneficial to obtain a bioactive, biointeractive flowable guttapercha sealer for wet apices with periapical defects. It was claimed that Guttaflow bioseal is able to create a three-dimensional root canal obturation with significantly fewer voids and gaps which produces a fluid tight seal of root canal systems [15, 16].

The aim of our study was to evaluate the efficiency of diode LASER activated irrigation in the removal of GuttaFlow Bioseal root canal filling material during re-treatment. The null hypothesis was rejected in this study, since the passive ultrasonic irrigation showed superior results regarding the removal of guttaflow bioseal.

The re-treatment procedure was considered complete when there was no evident filling material on the last instrument used. However, all the canals had remaining obturation material on the canal walls, which is in accordance with previous studies [17, 18]. This indicates that the absence of obturation material on the file does not correlate with complete removal of the filling material from the canal.

The amount of remaining filling obturation material was evaluated by longitudinal sectioning of the samples and quantitative analysis [1]. The sectioning of roots must be performed carefully so as not to dislodge the gutta-percha from the canal walls. However, one limitation of this method is calculating the remaining materials through two-dimensional view only which may influence the assessment outcome. Also, ImageJ software used for analysis is somewhat a subjective method for evaluation of the remaining filling material. Three different aspects of the tooth were evaluated: the apical, middle, and coronal thirds in one half of a split root specimen. It was reported that this method was effective in determining the amount of filling residue and minimized subjectivity [19].

In the present study, our results showed that the CPI samples had more residual remaining material than the other groups. The LAI group and the PUI group showed better removal of root canal filling material along all the root canal thirds. The LASER Activated group had more residual remaining filling material than the passive ultrasonic group which had the least remaning material in the three groups [20]. The use of passive ultrasonic activation is well-accepted and has been reported to improve the overall cleaning of the root canal system during endodontic retreatment [21–23]. The use of LASER activated irrigation also improved the removal of the residual root canal filling materials [24, 25].

The better effect of PUI and LAI on removal of the residual canal filling material may be related to their mechanism. PUI produces a rapid circular and swirling motion in the irrigant, causing an acoustic flow and inducing a cavitation effect around the ultrasonic file [26]. Similarly, the LASER irradiates the irrigant, with subsequent irrigant vaporization, resulting in the formation of vapor bubbles. These bubbles expand and implode with cavitation effects. The irrigant rushes into the bubble from the back, so the imploding bubble become sickle-shaped [27, 28].

LAI and PUI were superior to CPI irrigation in removing residual material from the apical third of the canal. This finding indicated that LAI and PUI performed effectively as additional techniques after the use of NiTi instrumentation in endodontic re-treatment to remove the residual material in the apical canal [29].

In this Study, none of irrigation activation techniques used during re-treatment could completely remove filling from the root canal walls. These findings are in agreement with those found in many other studies [1, 4, 19, 30–32].

Our results showed that a greater percentage of obturation material was found in the apical third than in middle and coronal thirds in all groups. This might be attributed to the anatomical variations that are often greatest in the apical third of the root canal [33]. Thus, the used re-treatment files, when rotating, shape the root canal into a form that has a rounded cross section, not reaching the canal irregularities leaving residual material on the canal walls [1]. These results are in full agreement with the previous studies. Somma et al. found that the remaining obturation material was found mostly in the apical third of the canal than the middle and coronal thirds [34].

Previous studies showed that the GuttaFlow 2 was removed significantly better from the canal walls than gutta-percha and resin-based sealers regardless of the rotary instrument system used [35]. This might be attributed to inability of GuttaFlow 2 to chemically bond to the canal walls and was frequently “peeled off” in the canal during re-treatment in previous studies [35, 36]. This has been modified in Guttaflow Bioseal which showed superior hydrophilicity and flowability with bioactive properties to achieve a three-dimensional root canal obturation with a predictable option for re-treatment which is considered a remarkable advantage.

### Study limitations

The limitations of this study were the variation in the root canal cross-sections between different premolars might affect the removal of root canal filling materials despite attempts to standardize the roots. Another limitation was the subjectivity of the ImageJ software in measuring the amount of remaining root canal filling materials. However, 3 different interpreters collected the data to minimize the subjectivity of this process. Also, the inability to differentiate between sealer and guttapercha using the software as the color map shows both with the same color gradient. The scientific researches related to the retrievability of the GuttaFlow Bioseal are almost non-existent so the comparison was not applicable.

## Conclusion

Under the circumstances of this study, it can be concluded that:

1. The use of irrigation activation technique is mandatory to maximize the amount of removed material.
2. No technique neither instrument, can completely remove the root canal obturation material.
3. The GuttaFlow Bioseal sealer has a very good retrievability leaving minimum amount of obturation material as long as potent activation of irrigation method is used.
4. The use of diode LASER was less effective in removal of root canal filling material than the PUI.

## Recommendations

1. Further studies should be done using different techniques of activation of irrigation using other types of LASERs and combining several methods of activation.
2. Other studies should be carried out on molars, investigating retrievability of root canal filling material in different cross sections of root canals.
3. Different imaging techniques could be used to measure the amount of remaining root canal filling materials.
4. Further in-vivo studies need to be done to correlate with the present study.

## Data Availability

The data presented in this study are available upon request from the corresponding author.
